# Towards Semantic e-Science for Traditional Chinese Medicine

**DOI:** 10.1186/1471-2105-8-S3-S6

**Published:** 2007-05-09

**Authors:** Huajun Chen, Yuxin Mao, Xiaoqing Zheng, Meng Cui, Yi Feng, Shuiguang Deng, Aining Yin, Chunying Zhou, Jinming Tang, Xiaohong Jiang, Zhaohui Wu

**Affiliations:** 1 College of Computer Science, Zhejiang University, Hangzhou, 310027, P.R. China; 2 China Academy of Traditional Chinese Medicine, Beijing, 100700, P.R. China

## Abstract

**Background:**

Recent advances in Web and information technologies with the increasing decentralization of organizational structures have resulted in massive amounts of information resources and domain-specific services in Traditional Chinese Medicine. The massive volume and diversity of information and services available have made it difficult to achieve seamless and interoperable e-Science for knowledge-intensive disciplines like TCM. Therefore, information integration and service coordination are two major challenges in e-Science for TCM. We still lack sophisticated approaches to integrate scientific data and services for TCM e-Science.

**Results:**

We present a comprehensive approach to build dynamic and extendable e-Science applications for knowledge-intensive disciplines like TCM based on semantic and knowledge-based techniques. The semantic e-Science infrastructure for TCM supports large-scale database integration and service coordination in a virtual organization. We use domain ontologies to integrate TCM database resources and services in a semantic cyberspace and deliver a semantically superior experience including browsing, searching, querying and knowledge discovering to users. We have developed a collection of semantic-based toolkits to facilitate TCM scientists and researchers in information sharing and collaborative research.

**Conclusion:**

Semantic and knowledge-based techniques are suitable to knowledge-intensive disciplines like TCM. It's possible to build on-demand e-Science system for TCM based on existing semantic and knowledge-based techniques. The presented approach in the paper integrates heterogeneous distributed TCM databases and services, and provides scientists with semantically superior experience to support collaborative research in TCM discipline.

## Background

Traditional Chinese Medicine (TCM) is a medical science that reflects traditional Chinese culture and philosophical principles. As a kind of complex medical science, TCM embodies rich dialectical thought, puts the human body into a large system for observation and adjusts the relations among formations, factors and variables to remain in a healthy status. Recent advances in Web and information technologies with the increasing decentralization of organizational structures have resulted in massive amount of TCM information resources (literature, clinical records, experimental data, etc.). The vast amount of TCM information resources today is distributed among many specialized databases like medical formula database, electronic medical record database, clinical medicine database, and so on [1]. For example, the Consortium for Globalisation of Chinese Medicine (CG-CM) is a global non-profit organization, with a mission of advancing the field of Chinese herbal medicine to benefit human kind through joint efforts of the academic institutions, industries and regulatory agencies around the world. Members of CG-CM have TCM information resources of their own. Many TCM scientists and biologists begin to use bioinformatics methods to analyze TCM contents from different points like biochemistry, genetics and molecular biology, so more and more biology databases have been introduced into TCM research. The information in those databases is potentially related to each other within a TCM knowledge-based system, and it is necessary for TCM scientists to reuse them in a global scope. There is an increased emphasis on integration of heterogeneous information resources in present of such a new setting. TCM scientists need to perform dynamic data integration over hundreds of or even thousands upon thousands of geographically distributed, semantically heterogeneous data sources that are subject to different organizations.

Besides the emerging information resources like databases, many scientific methods and processes in TCM have been enclosed as services (e-learning services, information analysis services, data mining services, etc.) by different organizations. There are many bioinformatics services available on-line, which TCM scientists can use to improve their research from the point of biology. Service-oriented science [2] has the potential to increase individual and collective scientific productivity by making powerful information tools available to all and thus enabling the widespread automation of data analysis and computation. Scientists and applications are able to access Web services to finish specific tasks or gain information. As scale increases, creating, operating, and even accessing services become challenging. Services are designed to be composed under some contexts, that is, combined in service workflows to provide functionality that none of the component services could provide alone. There is an increased requirement on coordination of various TCM services to support collaborative and on-demand scientific activities.

E-science is the term applied to the use of advanced computing technologies to support global collaboration for scientists [3]. However, complete and seamless TCM e-Science is impeded by the heterogeneity and distribution of the independently designed and maintained information and service resources. The use of domain knowledge provides a basis for full interoperability in a distributed environment like the Web. As the foundation of the Semantic Web [4], ontologies [5] are the specification of conceptualisations, used to help programs and humans share knowledge. Encoding domain knowledge in terms of ontologies provides a possible approach to overcome the problem of semantic heterogeneity of both information and service resources. As before-mentioned, there are many information resources in the TCM discipline and most of them exist in terms of databases. Formal semantics in ontologies has provided a feasible way to integrate scientific information resources in a conceptual information space. Besides, some semantic mark-up languages like OWL-S [6] are used to describe services with more precise semantics. Richer semantics helps to provide automation or semi-automation of such activities as verification, simulation, configuration, composition, and negotiation of services. From this point of view, the research in knowledge-based approaches especially the semantic techniques has pointed out a new direction to realize the vision of e-Science for TCM.

A number of approaches for e-Science in biology or medicine have been proposed or developed. Stevens et al. in [7] aim to exploit Grid techniques especially the Information Grid to achieve e-Science for bioinformatics. They present the myGrid platform that provides middleware layers to satisfy the needs of bioinformatics. The myGrid platform is building high level services for data and application integration such as resource discovery, workflow enactment and distributed query. Tao et al. in [8] illustrate through a Semantic Web based knowledge management approach the potential of applying Semantic Web techniques in an e-Science pilot project called GEODISE for the domain of Engineering Design Search and Optimization. They design advice mechanisms based on semantic matching, to consume the semantic information and facilitate service discovery, assembly and configuration in a problem solving environment. They have shown the potential of using semantic technologies to manage and reuse knowledge in e-Science. Roure et al. in [9] analyse the state of the art and the research challenges involved in developing the computing infrastructure for e-Science. They propose the future e-Science research infrastructure, which is termed the Semantic Grid and a conceptual architecture for the Semantic Grid is presented, which adopts a service-oriented perspective. They consider the requirements of e-Science in the data/computation, information and knowledge layers. Clearly, e-Science is a widely open research area and there is still much room for improvement on all existing approaches, especially for achieving on-demand e-Science in knowledge-intensive domain like TCM.

In this paper, we address the before-mentioned issues by applying semantic techniques and standards such as RDF [10] and OWL [11] to enable database integration and service coordination towards the full richness of e-Science vision of TCM science over the Internet. To achieve this vision, we propose an approach (1) to model on domain knowledge and develop large-scale domain ontology (2) to interconnect distributed databases using richer semantics as one huge virtual database, and (3) to coordinate scientific services by semantic-driven workflow. We present a dynamic and extendable approach to build on-demand e-Science applications for knowledge-intensive disciplines like TCM based on the semantic techniques. We recognize TCM research as information gathering and process workflows. We have designed and developed the approach as a layered structure to satisfy the TCM research requirements in e-Science. The proposed methods aim at facilitating the integration and reuse of distributed TCM database and service resources in cyberspace, and deliver a semantically superior experience to TCM scientists. We have developed a collection of semantic-based toolkits to facilitate TCM scientists and researchers in information sharing and collaborative research.

## Results

### System architecture

**Figure 1:**
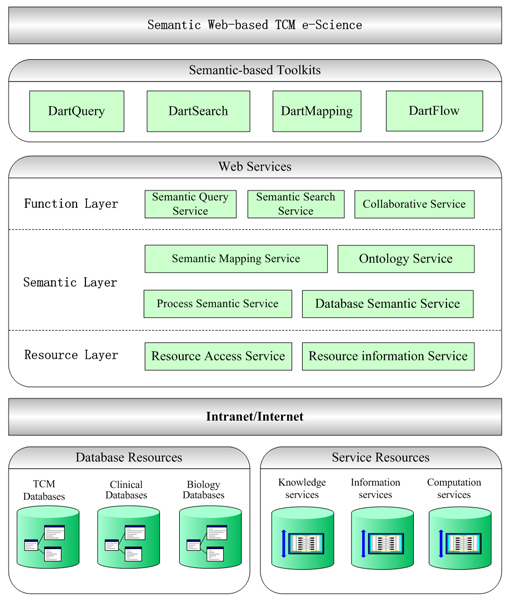
**Architecture**. The abstract architecture of Semantic Web e-Science for TCM.

Briefly, we illustrate the abstract architecture of our approach in figure 1. In our approach, a TCM e-Science system is composed of client side and server side. We have designed and developed the server side as a layered structure including resource layer, semantic layer and function layer.

• The resource layer mainly supports the typical remote operations on the contents of resources on the Web and querying the meta-information of databases and services. The services in this layer extend some core Grid [12,13] services from the Globus [14] platform. We build the whole e-Science system on these Grid services that provide the basic communication and interaction mechanism for TCM e-Science. There are two services in this layer. *Resource Access Service *supports the typical remote operations on the contents of databases and execution of services. To relational databases, the operations contain query, insertion, deletion, and modification. *Resource Information Service *supports inquiring about the meta-information of database or service resources including: relational schema definition, DBMS descriptions, service descriptions, privilege information, and statistics information.

• The semantic layer is mainly designed for semantic-based information manipulation and integration. This layer is composed of two sub-layers. The lower layer contains two services. *Process Semantic Service *is used to export services as OWL-S descriptions. *Database Semantic Service *is used to export the relational schema of databases as RDF/OWL semantic description. The upper layer contains two services. *Ontology Service *is used to expose the shared TCM ontology and provide basic operations on the ontology. Ontology is used to mediate and integrate heterogeneous databases and services on the Web. *Semantic Mapping Service *establishes the mappings from local resources to the mediated ontology. Semantic Mapping Service maintains the mapping information and provides the mechanism of registering and inquiring about the information.

• The function layer delivers a semantically superior experience to users to support scientific collaborative research and information sharing. *Semantic Query Service *accepts semantic query, inquires Semantic Mapping Service to determine which databases are capable of providing the answer, and then rewrites the semantic queries in terms of database schema. A semantic query is ultimately converted into a set of SQL queries. The service wraps the results of SQL queries by semantics and returns them as triples. *Semantic Search Service *indexes all databases that have been mapped to mediated ontology and accepts semantic-based full-text search. The service uses the standard classes and instances from the TCM ontology as the lexicon in establishing indexes. *Collaborative Service *discovers and coordinates various services in a process workflow to supports research activities in a virtual community for TCM scientists.

Note that we differentiate two kinds of services. The services in this architecture are fundamental services to support the whole e-Science system, whereas there are many common services treated as Web resources for e-Science process. At the client side, the e-Science system provides a set of semantic-based toolkits to assist scientists to perform complex tasks during research. We call this architecture *Dart *(Dynamic, Adaptive, RDF-mediated and Transparent) [15], which is an abstract model for TCM e-Science. A detailed description of the service-oriented architecture is provided in the Methods section.

### TCM domain ontology

Recent advent of the Semantic Web and bioinformatics has facilitated the incorporation of various large-scale online ontologies in biology and medicine, such as UMLS [16] for integrating biomedical terminology, Gene Ontology [17] for gene product and MGED Ontology [18] for microarray experiment. As the backbone of the Semantic Web for TCM, a unified and comprehensive TCM domain ontology is also required to support interoperability in TCM e-Science. To overcome the problem of semantic heterogeneity and encode domain knowledge in reusable format, we need an integrated approach to develop and apply a large-scale domain ontology for the TCM discipline. In collaboration with the China Academy of Traditional Chinese Medicine (CATCM), we have taken more than 5 years in building the world's largest TCM domain ontology [19].

**Figure 2:**
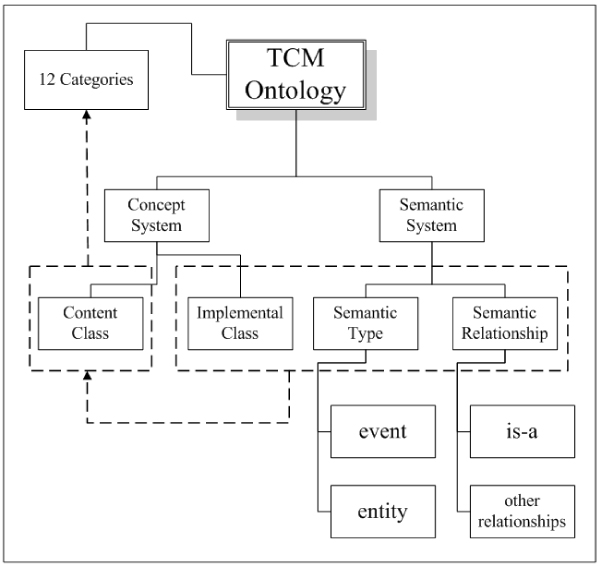
**Semantic system framework**. The semantic system framework of the TCM ontology.

We divide the whole TCM domain into several sub-domains. The TCM ontology is developed collaboratively by several branches of the CATCM as *categories*. A category is a relatively independent ontology corresponding to a relatively closed sub-domain, compared with the ontology corresponding to the whole domain. There are 12 categories in the current knowledge base of the TCM ontology. Each category is corresponding to a sub-domain (Basic Theory of TCM, Formula of Herbal Medicine, Acupuncture, etc) of TCM. We list the characterization of content of each category in table 1. Considering medical concepts and their relationships from the perspective of TCM discipline, we define the knowledge system of the TCM ontology by two components: concept system and semantic system (see figure 2). The concept system contains *content classes *that represent the domain knowledge of the TCM discipline and 4 kinds of basic implemental classes (name class, definition class, explanation class, and relation class) to define each content class. The semantic system concerns the basic semantic type and semantic relationship of class. A class has literal property and object property. The range of a literal property is a literal or string, whereas the range of an object property is a class. A content class has 5 object properties (see table 2) with each related with a class. Relation class has two properties: the range of the former is semantic relationship and the range of the latter is content class. Content classes are related with each other through semantic relationship. In this way, all content classes in the TCM ontology have the unified knowledge structure whereas different instances of content class have various contents and relationships.

There are more than 20,000 classes and 100,000 instances defined in the TCM ontology and the ontology has become a distributed large-scale knowledge base for TCM domain knowledge. The ontology has become large enough to cover different aspects of the TCM discipline and is used to support semantic e-science for TCM. As a large-scale domain ontology, the TCM ontology is used to integrate various database resources in a semantic view and provide formal semantics to support service coordination in TCM e-Science.

### TCM database integration

We use ontology semantics to integrate distributed TCM databases as a global virtual database. We have developed a set of semantic-based toolkits for scientists to integrate and use information in distributed TCM databases.

### DartMapping

**Figure 3:**
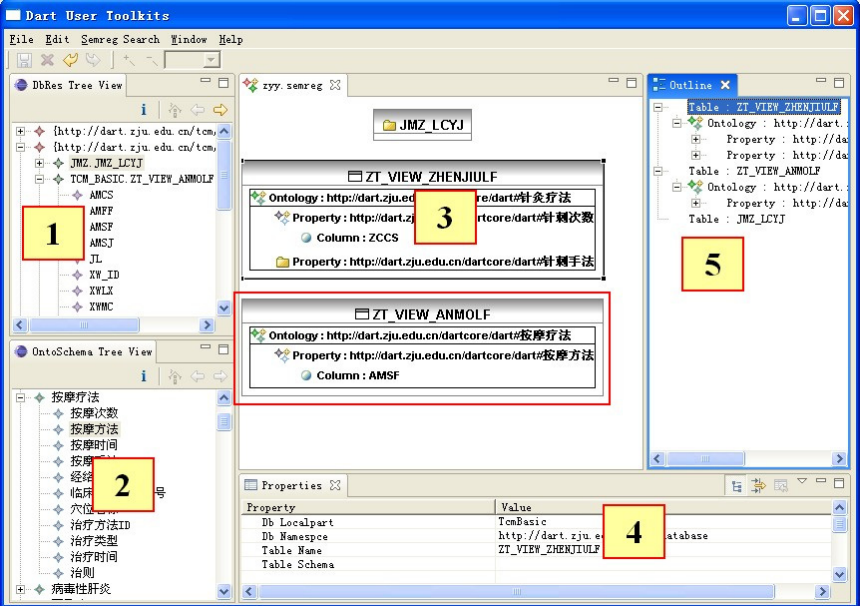
**DartMapping**. The default user interface for DartMapping.

In our approach, before-mentioned domain ontology acts as a semantic mediator for integrating distributed heterogeneous databases. Relational schemata of distributed TCM databases are mapped to the TCM ontology according to their intrinsic relations. To facilitate the process of *semantic mapping *between the schemata of local databases and the semantics of the mediated ontology, we have developed a visual semantic mapping tool called *DartMapping *for integrating relational databases in a Semantic Web way (see figure 3). The tool provides two major functions: establishing semantic mapping from heterogeneous relational database to a mediated ontology semi-automatically, especially mapping for composite schema with complex join between tables, and converting relational databases schema to ontology statements based on the semantic mapping information.

Figure 3 depicts how we use DartMapping to establish mapping between ontology and database schema. Relational database schema is displayed in hierarchy including the names of databases, tables and the corresponding fields (1). The class hierarchy and class properties of the mediated ontology are displayed below (2). Classes and properties are displayed as labels in the panel. User drags tables and classes into the main panel (3), and establishes their mappings directly. One table is likely to be mapped to more than one class. The meta-information about the selected table is shown under the main panel (4). The right panel shows the outline of the mapping definitions (5). A mapping definition can be exported as XML files and reused by applications. Besides, users are able to query mapping information defined previously in DartMapping. TCM scientists are able to map local databases to the mediated TCM ontology with DartMapping. Distributed and heterogeneous databases including TCM databases (e.g. herbal medicine formula database), clinical databases (e.g. EHR database) and biology databases (e.g. neuron database) are integrated as knowledge sources for TCM scientists to carry out research. TCM scientists are able to perform searching and querying over the integrated databases to gain useful information in research.

### DartSearch

We developed a database search engine called *DartSearch *to enable full-text search over distributed databases. Scientists are able to perform searching through the integrated databases to get required information as we do in search engines like Google [20]. However, search here is different with Google-like search. The search process is performed based on the semantic relations of the ontology. We call it *semantic search*, which is searching for data objects rather than Web pages. Semantics is presented in two aspects in DartSearch:

• We construct a domain-specific lexicon for segmentation based on the TCM ontology. Each term in the lexicon is a class or instance in the ontology plus its part of speech. When we segment a piece of information from database, only the words that appear in the TCM ontology are segmented whereas other words are discarded as irrelevant information to TCM semantic search.

• Unlike keyword-based index in traditional search engine, we construct index for classes or instances in databases. The semantic relations between those classes or instances are encoded as part of the index.

In this way, scientists are able to search with more accurate constraints and get more relevant information from search results. For example, if a TCM scientist wants to find some TCM formulas that cure *influenza*, then he can use influenza as a keyword to perform a semantic search. The search returns TCM-specific information and the information that doesn't contain the keyword influenza but contains terms related to influenza is also returned. We connect directly-matched information and relevant information by using semantic relations in the ontology.

**Figure 4:**
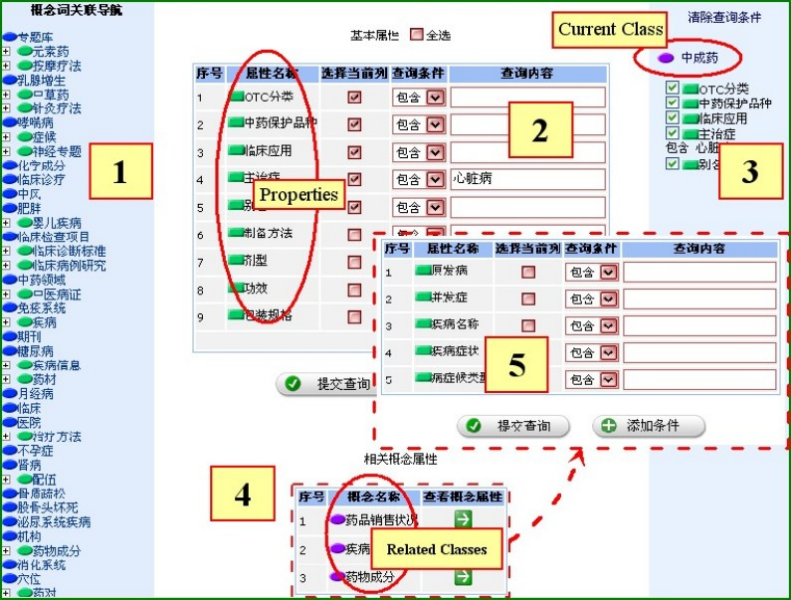
**DartSearch**. The default user interface for DartSearch.

We provide users with a Google-like search interface to perform semantic search (see the bottom left panel in figure 4) in DartSearch. The result of a semantic search request is shown in Figure 4 with *gene *used as the keyword. The statistics information about the search result (e.g. the number of items) is displayed (1). DartSearch lists the items in a descending order according to their matching degrees to the keywords of the search (2). Each item in the list is a piece of information from databases that have been mapped to TCM ontology classes (3). At the bottom of each item there are the classes the item is mapped to (4), the classes relevant to the mapping classes (5) and the matching degree to the keyword. The classes and relevant classes are connected by semantic relations in the ontology. The schemata of a database are allowed to be mapped to several categories of the TCM ontology. Categories that relevant to the search result are listed in a descending order according to their matching degrees to the search (6).

### DartQuery

**Figure 5:**
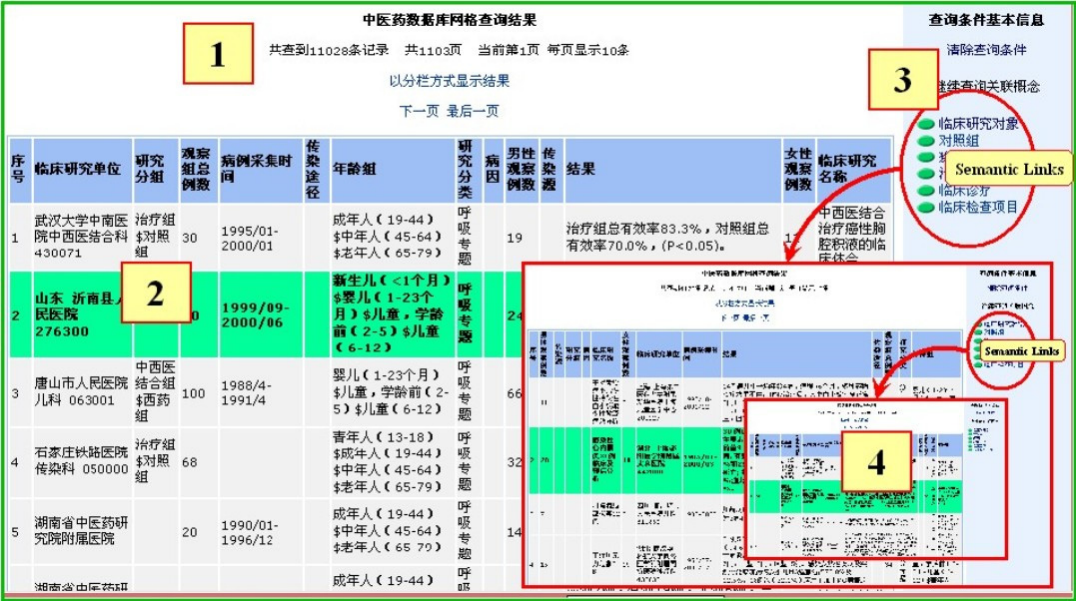
**DartQuery**. The default user interface for DartQuery.

Generally, semantic search only gives us a coarse set of result. If scientists want to get more exact information, they are able to perform querying instead of searching in the semantic layer. A Web-based query tool called *DartQuery *is provided for scientists to query over distributed TCM databases dynamically (see figure 5). Relevant categories generated during semantic search imply the possible scopes from within scientists perform semantic query. They are able to select the category with the largest matching degree to construct a semantic query statement. To enable querying in the semantic layer, we use the SPARQL [21] query language. Every query in SPARQL is viewed as an ontology class definition, and processing a query request is reduced as computing out ontology instances satisfying the class definition [22]. The statement of a semantic query about the properties (name, usage, composition, etc.) of a TCM formula that cures influenza is as follows:

SELECT ?fn ?fu ?fc ?dn ?dp ?ds

WHERE {

   ?y1 rdf : type tcm : Formula_of_Herbal_Medicine

   ?y1 tcm : name ?fn

   ?y1 tcm : usage_and_dosage ?fu

   ?y1 tcm : composition ?fc

   ?y1 tcm : cure ?y2

   ?y2 tcm : name "influenza"

   ?y2 tcm : pathogenesis ?dp

   ?y2 tcm : symptom_complex ?ds

}

Such a query in SPARQL is constructed dynamically. A form-like query interface is used to facilitate users in constructing semantic query statements in web browser. The user interface incorporates an open-source AJAX framework [23], which enables immediate data update without refresh Web pages in web browsers. DartQuery generates querying forms automatically according to the class definitions in a category. Scientists who want to query something are able to construct a query statement by selecting classes and properties from the forms in the query interface. Figure 5 depicts the process how a user constructs a semantic query about **traditional patent medicine**. Starting from the ontology view panel on the left, user is able to browse the hierarchy tree and select the relevant classes (1). A query form corresponding to the property definitions of the selected class is automatically generated and displayed in the middle. User could select properties of interest, and inputs query constraints such as the **efficiency **of the medicine (2). An outline of the currently-built query including the current class is displayed (3). User could further explore into the classes related (e.g. **disease**) to the current one, and construct more complex semantic queries spanning over several classes (4). User is led into the query interface of related classes, and could add more query constraints (5).

**Figure 6:**
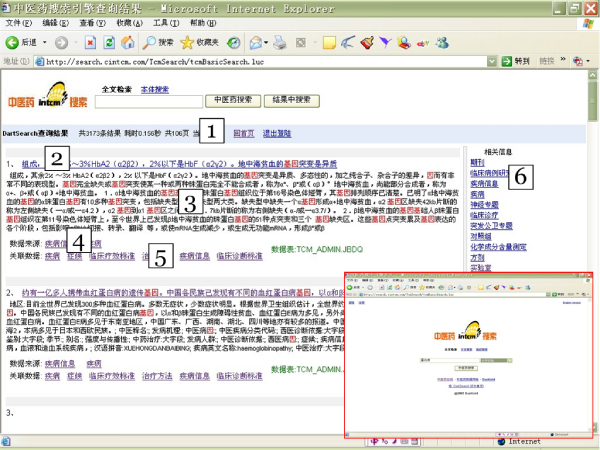
**Query result**. The query result of DartQuery.

The SPARQL query statement is submitted to the system and converted into a SQL query plan according to the mapping information between database schemata and the mediated ontology. The SQL query plan is then dispatched to specific databases for information retrieval. The query returns all satisfactory records from databases that have been mapped to the ontology. Since the query result from databases is just a record set without any semantics, the system converts the record set into a data stream in RDF/XML format and the semantics of the result is fully presented. Figure 6 depicts the situation in which a user is navigating the query result. The statistics information about the query result is displayed (1). User selects one data object, which is highlighted (2). By following semantic links, user could get all those data objects semantically related to the current one (3). Note that the relations between the selected object and those discovered by following semantic links are derived from the ontology in the semantic layer. User could keep on navigating through a collection of databases as long as they are semantically connected (4).

### TCM service coordination

Ontology semantics are used to support dynamic and on-demand service coordination in a VO. Scientists are able to discover, retrieve and compose various services to achieve complex research tasks in a visual environment.

### Knowledge discovery service

There are various services in a TCM VO and we mainly recognize 3 kinds of services: computation services, information services, and knowledge services. Computation services are services that execute computational jobs or analyze scientific data. Information services are services that manipulate and provide specific information. Semantic query service and semantic search service mentioned-before are 2 typical information services. Knowledge services are services that apply information to solve domain-specific problems or discover facts. Different services are used to support different kinds of tasks for TCM research.

One of the most important knowledge services for TCM research is the knowledge discovery service. The distributed databases integrated under the ontology contain much implicit domain knowledge that is hard to be discovered manually by human-being and thus require some intelligent methods to assist scientists to discover the implicit knowledge. For example, a formula of herbal medicine is composed of several individual drugs. In database of herbal medicine formula, we get the components of a formula directly; however, the same individual drug may appear in several formulas, and then the correlation between two individual drugs in various formulas can't be acquired directly by querying or searching. Notice that, according to TCM theory, a relatively fixed combination of several individual drugs is called a paired-drug when such a combination is able to strengthen their medical effects, or lessen the toxicity and side effects of some drugs. Implicit knowledge such as "paired-drug" is more likely to be discovered by data mining, instead of directly querying or searching information resources. Our method integrates several semantic-based data mining algorithms like the associated and correlated pattern mining [24] to achieve knowledge discovery on distributed databases. Scientists are able to select knowledge discovery service according to the requirements of the research task and perform knowledge discovery over a selective set of information from distributed databases.

### DartFlow

**Figure 7:**
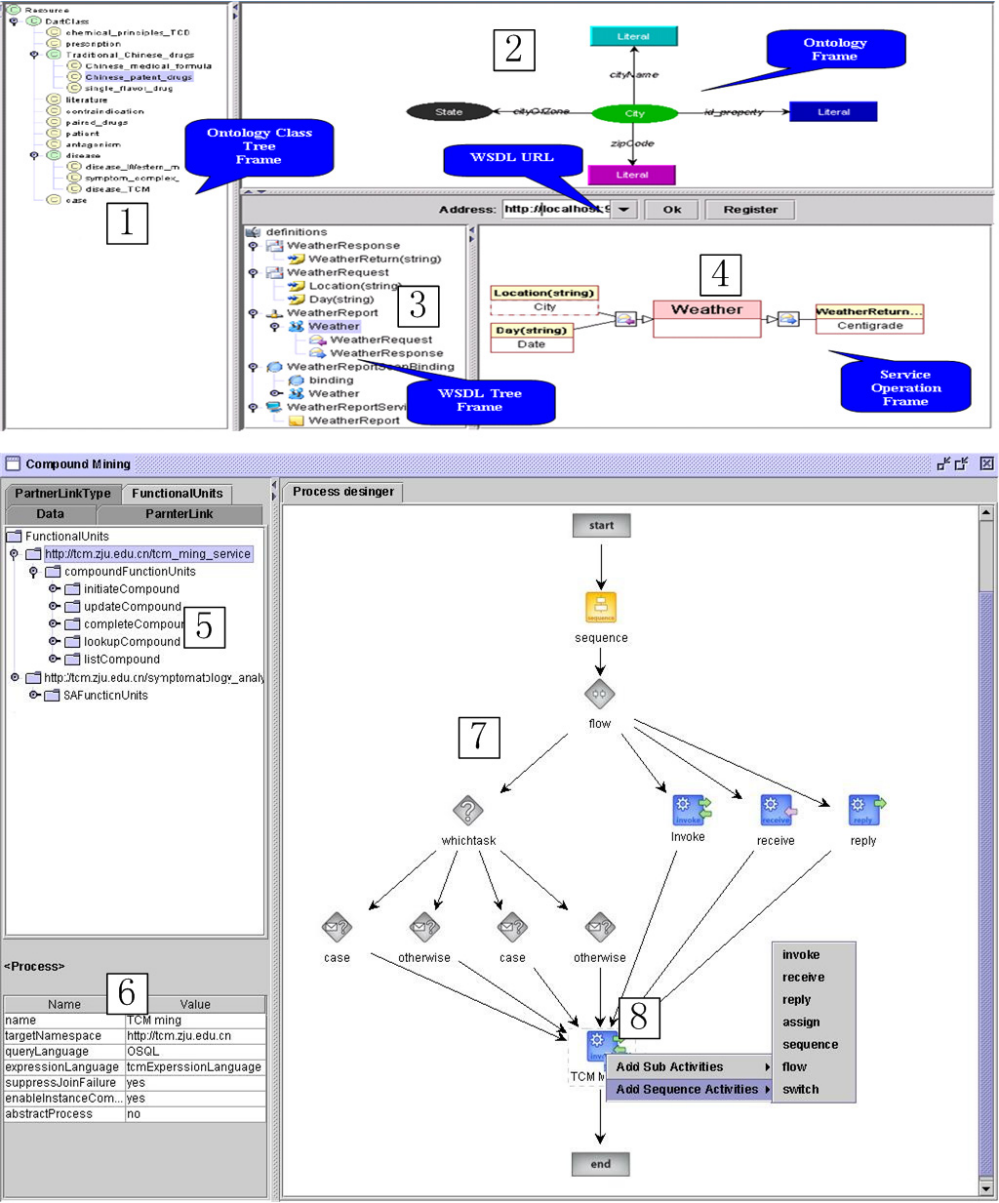
**DartFlow**. The user interface of DartFlow.

Besides database integration, a sophisticated e-Science system should also support service coordination for scientists, which is a significant part of TCM e-Science. Similarly with bioinformatics, TCM scientific research often requires coordination and composition of service resources. We have applied semantic techniques to achieve dynamic and on-demand service coordination in a VO and developed a Web-based service coordination tool (see figure 7) called *DartFlow *[25]. DartFlow provides a convenient and efficient way for scientists to collaborate with each other in research activities. It offers interfaces to allow researchers to register, query, compose and execute services in the semantic layer.

Service providers register component web service into the VO before service composition. DartFlow integrates a service registration portal for scientists to register new services. The class hierarchy (1) and class properties (2) of the mediated ontology are displayed graphically. Service description (e.g. the input and output parameters) is displayed in hierarchy (3). Similarly with semantic mapping in database integration, service providers create mappings between ontology classes and service descriptions (4). The mapping information is stored in the repository of the portal. Automatic service discovery and service matchmaking is achieved based on semantics. So far DartFlow has been full of a collection of scientific services, which are all provided by different TCM research institutes.

When a VO has been filled with various applied service, scientists are able to build serviceflow to achieve complex research tasks in DartFlow. We should retrieve enough services in order to compose a serviceflow. If scientists want to query services, they submit a service profile (e.g. a service to analyze TCM clinical data) to the portal specifying their requirements. The portal invokes suitable matchmaking agent to retrieve target services for users (5). The agent has been implemented according to some semantic-based service matchmaking algorithm. Scientists are able to compose retrieved services (6) into a serviceflow in the workspace (7) to achieve a research task. In order to enhance the flexibility and usability of serviceflow, DartFlow supports both static activity node and dynamic activity node in serviceflow (8): the former refers to those nodes combined with specific applied services at build-time; and the latter refers to those nodes combined with semantic information. After a serviceflow is designed graphically, the corresponding OWL-S file is generated according to the service mapping information. Scientists are able to validate the serviceflow from both logic aspect and syntax aspect with a validator in DartFlow and the validated serviceflow will be executed ultimately.

### TCM collaborative research scenario

**Figure 8:**
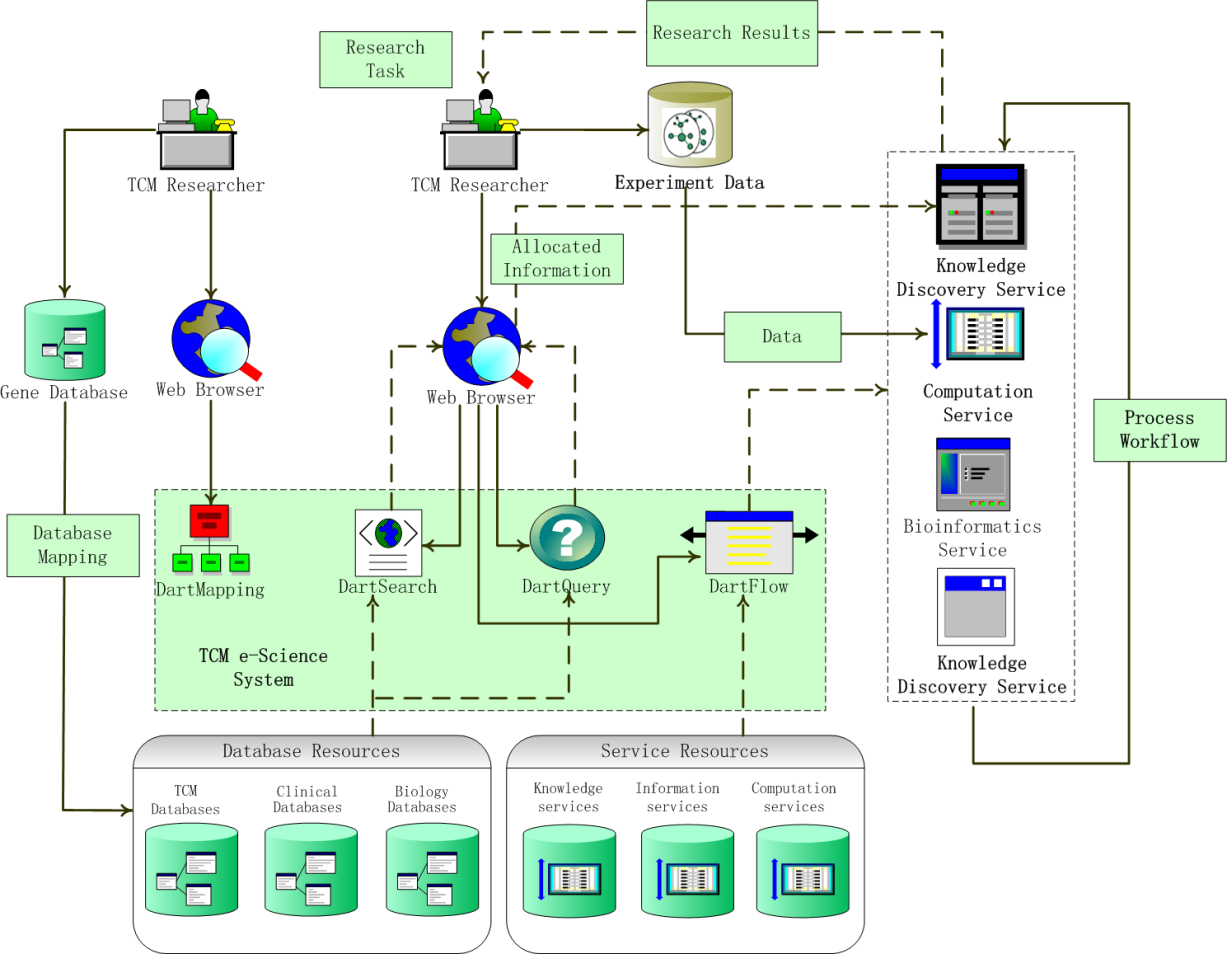
**Scenario**. A scenario about TCM collaborative research based on the TCM e-Science system.

The proposed semantic-based approach is able to support TCM scientists to perform research collaboratively in a VO. TCM scientists are able to use the semantic-based toolkits before-mentioned in web browsers anywhere to solve problems and finish tasks. We illustrate the application of our approach through the following collaborative research scenario as several steps (see figure 8):

### Task-driven information allocation

Information resources are often related to perform a research task. Grouping task-related information resources is a precondition for achieving collaborative research. Given a research task, TCM scientists are able to perform semantic search or construct semantic query to allocate information according to the task context. A TCM scientist, say, Wang is performing a research task about *the impact of herbal medicine formula on gene expression*. As a TCM scientist, Wang is not familiar about biology especially gene, so he needs to find some initial information about gene before starting to conduct experiments. He is able to perform semantic search over distributed databases in DartSearch about gene as well as its relations with TCM. DartSearch will return a general result about gene and its relation with TCM. If Wang wants more exact results about current research progress about herbal medicine formula and gene, he can perform semantic query in DartQuery. The semantic search in DartSearch has implied that the required information is mainly located in categories Formula of Herbal Medicine and Diseases. Then Wang is able to perform semantic query within the databases that has been integrated under these two categories. Wang constructs semantic query statements dynamically in DartQuery and the query returns a collection of literature about herbal medicine formula and gene.

### Collaborative information sharing

After reading a batch of relevant papers, Wang decides to perform further research about the relations between herbal medicine formula and gene expression. However, he finds the information he allocated is insufficient for his task, and it means the TCM VO lacks the required information. Scientists are able to allocate only a very small sub-set of information or services in the field of TCM. It's impossible for a single scientist to deal with all the domain information. Scientists can share information collaboratively in a VO based on the semantic e-Science system. Wang can communicate with other scientists in the VO to ask for required information. Fortunately, an institute in the VO has developed a new database that contains information about gene expression. The institute registers the database into the VO by creating semantic mappings with DartMapping. Then Wang is able to get further information about gene expression by querying the database.

### Scientific service coordination

Wang selects suitable services according to his research requirements and designs a serviceflow in DartFlow to achieve his research goal (see figure 8). The first knowledge discovery service in the serviceflow is used to discover some underlying rules from the allocated information. The result of knowledge discovery has shown that there exists underlying relation between Sini decoction (a kind of herbal medicine formula) and glutathione S-transferase (GST) gene expression from many research papers. Wang starts to conduct experiments on the impacts of Sini decoction on GST gene expression. The experiment data is submitted to the computation service in the serviceflow. He also uses bioinformatics services such as BLAST in the serviceflow to deal with the works related with GST gene. The final result of the serviceflow has shown that *Sini decoction has strong impacts on GST gene expression*. The serviceflow here may involve a recursive process in order to refine the result.

## Discussion

Due to the bottleneck of information extraction and NLP, the proposed approach is inclined to structured information resources rather than unstructured or semi-structured resources. However, much information is involved in those resources, which we can't integrate into the TCM e-Science system well with the current method. Although we could extract schemata from unstructured or semi-structured resource and map those to the mediated ontology in a similar way as database integration, there leaves much work to be done for the purpose. We have provided a set of semantic-based toolkits to assist TCM scientists to reuse information and carry out research. Although the tools are implemented and used based on web browser, the process of interaction may be still a little bit complex to TCM scientists who have no knowledge of semantics. As TCM is a traditional science and there are also many TCM scientists who are even not familiar with computer and Internet. To those scientists, we should improve the usage and convenience of the system to satisfy their requirements well.

## Conclusion

We have presented a comprehensive and extendable approach that is able to support on-demand and collaborative e-Science for knowledge-intensive disciplines like TCM based on semantic and knowledge-based techniques. The semantic-based e-Science infrastructure for TCM supports large-scale database integration and service coordination in a virtual organization. We have developed a collection of semantic-based toolkits to facilitate TCM scientists and researchers to achieve information sharing and collaborative research. We illustrate the application of the proposed approach through a TCM collaborative research scenario. Based on the proposed approach, we have built a fundamental e-Science platform for TCM in CATCM and the system currently provides access to over 50 databases and 800 services in practice. The result has shown that integrating databases and coordinating services with a large-scale domain ontology is an efficient approach to achieve on-demand e-Science for TCM and other similar application domains, such as life science and biology.

## Methods

### TCM ontology engineering

The TCM ontology is a basic element to achieve semantic e-Science for TCM, therefore the quality of the ontology directly affects the e-Science. We should develop the TCM ontology according to some criteria based on the agreement among the participant institutes. The development of ontologies is a modelling process that needs the cooperation of ontology engineers (also called ontologists) who should have sufficient understanding of the domain knowledge. To our experience, ontology construction is a complex and labor-intensive activity.

First, we employ a layered privilege model in ontology development. Users that play different roles in the process of ontology development hold different privileges. There are mainly four kinds of privileges: reader, editor, checker and administrator.

• Ontology readers are able to browse all the contents of the ontology.

• Ontology editors are able to input, modify and delete instances within a category but have no privilege to manipulate the classes of the category.

• Ontology checkers own the privilege to manipulate both classes and instances in a category.

• Ontology administrators have the global privilege to all categories of the whole ontology.

Then, we could develop the ontology according to the following procedure:

• Analyze and determine knowledge sources. The scientific control of the conceptual glossary of a discipline is the most important issue in NLP. It's necessary to analyze specialized lexicons as the knowledge sources of ontology contents.

• Construct an upper-level conceptual framework. Comprehensive analysis and research of the disciplines is needed before the ontology design. A domain oriented conceptual framework is constructed to address all the knowledge engineering problems and instruct editing ontology content.

• Determine and assign developing tasks. Developing a large-scale ontology is a laborious work that requires collaborative efforts. We divide a large-scale ontology into categories and assign developing tasks to participants by category according to the complexity of each category.

• Extend conceptual hierarchy. Checkers create low-level class hierarchy.

• Materialize ontology contents. Editors extract and acquire domain knowledge from various sources, and formalize knowledge into instances.

• Check and revise contents. Checkers check each instance in the category they take charge of to make sure that there is no error or contradiction in newly input contents.

• Publish ontology by using user interface. The ontology is published as Web service and users are able to browse and query the ontology through Web browsers.

Step 4 to step 6 is a recursive process. Follow this general procedure and we are able to develop a large-scale domain ontology for e-Science system.

### View-based semantic mapping

The semantic-based e-Science system allocates database information and integrates heterogeneous databases together under the TCM ontology by creating semantic views. A relational table is mapped into one or more classes of the ontology and a table field is mapped to a class property. Implicit relationships between database resources are interpreted as semantic relations in the ontology.

According to the conventional data integration literature [26], view-based approach has a well-understood foundation and been proved to be flexible for heterogeneous data integration. There are two kinds of view in conventional data integration systems, GAV (global-as-view) or LAV (local-as-view). Considering the Semantic Web situation, GAV is to define each class or property as a view over relational tables, and LAV is to define each relational table as a view (or query) over the mediated ontology. The experiences from conventional data integration systems tell us that LAV provides greater extensibility than GAV: the addition of new sources is less likely to require a change to the mediated schema [26]. In the field of TCM, new databases are regularly added so total number of databases is increasing gradually.

Therefore, the LAV approach is employed in our method, that is, each relational table is defined as a view over the ontologies. We call such kind of views as *semantic view*, and such kind of mappings from relational database to ontology as *semantic mapping*. Like that in conventional data integration, a typical semantic view consists of two parts: the view head, which is a relational predicate, and the view body, which is a set of RDF triples. In general, the view body is viewed as a query over the ontology, and it defines the semantics of the relational predicate from the perspective of the ontology. The meaning of semantic view would be clearer if we construct a *target instance *based on the semantic mapping specified by these views. In this way, different TCM databases can be integrated under the shared TCM ontology. Scientists needn't care about the actual structure of database resources and they just operate on the semantic layer. More detailed aspects about semantic view and semantic mapping could be found in [27].

### Semantic-based service matchmaking

A service is abstracted as service description including input and out parameters. If the service descriptions are mapped to ontology classes, service matchmaking and composition can be achieved automatically and dynamically based on semantics. Ideally, given a user's objective and a set of services, an agent would find a collection of services requests that achieves the objective. We use a semantic-based method to achieve dynamic service matchmaking and composition in DartFlow. Assume X and Y are two ontology classes. We represent the matching degree of class X to class Y as Similarity(X, Y). If X can provide all the properties that Y embodies, they are totally matched. If X embodies Y, they are partially matched. The only partially provide the properties that value of Similarity(X, Y) ranges from 0 to 1. The value 0 means X is not semantically similar to Y at all, and the value 1 means X is the same as Y. Note that Similarity(X, Y) and Similarity(Y, X) represent different matching degrees. Different relations between X and Y result in different formulae of similarity evaluation.

Service matchmaking is to match a service request against a collection of services. As services are mapped to ontology classes by service providers, service matchmaking is reduced into calculating the semantic similarity of ontology classes. Given a service S = (I, O) and a service request R = (I_r_, O_r_), we can calculate the matching degree between S and R, which is denoted as Ω(S, R). Ω(S, R) is mainly determined by the similarity between I and I_r_, and the similarity between O and O_r_. Our algorithm ensures the value of a matching degree ranging from 0 to 1. Service composition is also performed based on semantic similarity. Given a service A = (I_a_, O_a_) in the serviceflow and a collection of candidate services, the service B = (I_b_, O_b_) will be selected as the subsequent service of A in the serviceflow as long as Ω(A, B) is the largest among the candidate services. More detailed aspects about the algorithm could be found in [25]. Given a representation of services as actions, we can exploit AI planning techniques for automatic service composition by treating service composition as a planning problem [28].

## Competing interests

The authors declare that they have no competing interests.

## Authors' contributions

The semantic-based approach was developed jointly by all authors and implemented by YM, HC, CZ, JT and SD. HC, YM, XZ, YF and SD have designed the system architecture, developed general concepts, participated in the manuscript writing and coordinated the study. The TCM ontology was mainly designed by HC, MC, AY and YM. The database integration method was mainly designed and developed by HC, YM, JT and CZ. The service coordination method was mainly developed by SD, HC and YM. The knowledge discovery method was mainly designed and developed by YF. All authors read and approved the final version of the manuscript.

## Figures and Tables

**Table 1:** TCM ontology categories. The initial categories defined in the TCM ontology corresponding to the sub-domains of TCM.

**Categories**
The Basic Theory of Traditional Chinese Medicine
The Doctrines of Traditional Chinese Medicine and Relevant Science
Chinese Materia Medica (Herbal Medicine)
Formula of Herbal Medicine
Humanities
Medicinal Propagation and Other Resources
Cause and Mechanism of Disease and Diagnosis
Therapeutic Principles and Treatments
Informatics and Philology
Acupuncture
Prevention
Diseases

**Table 2:** Content class structure. The structure of content class in the TCM ontology.

**Class Property**	**Property Value**	
hasNames	Name Class	
hasDefinitions	Definition Class	
hasExplanations	Explanation Class	
hasRelatedClasses	Relation Class	Semantic Relationship
		Content Class
hasSemanticTypes	Semantic Type	
